# Compact Shape Morphing Tensegrity Robots Capable of Locomotion

**DOI:** 10.3389/frobt.2019.00111

**Published:** 2019-11-01

**Authors:** Tyler Rhodes, Clayton Gotberg, Vishesh Vikas

**Affiliations:** Agile Robotics Lab (ARL), Department of Mechanical Engineering, University of Alabama, Tuscaloosa, AL, United States

**Keywords:** tensegrity, shape morphing, robot locomotion, tensegrity robot, tensegrity mechanism, tensegrity fabrication, sphericon, icosahedron

## Abstract

Robustness, compactness, and portability of tensegrity robots make them suitable candidates for locomotion on unknown terrains. Despite these advantages, challenges remain relating to ease of fabrication, shape morphing (packing-unpacking), and locomotion capabilities. The paper introduces a design methodology for fabricating tensegrity robots of varying morphologies with modular components. The design methodology utilizes perforated links, coplanar (2D) alignment of components and individual cable tensioning to achieve a 3D tensegrity structure. These techniques are utilized to fabricate prism (three-link) tensegrity structures, followed by tensegrity robots in icosahedron (six-link), and shpericon (curved two-link) formation. The methodology is used to explore different robot morphologies that attempt to minimize structural complexity (number of elements) while facilitating smooth locomotion (impact between robot and surface). Locomotion strategies for such robots involve altering the position of center-of-mass (referred to as internal mass shifting) to induce “tip-over.” As an example, a sphericon formation comprising of two orthogonally placed circular arcs with conincident center illustrates smooth locomotion along a line (one degree of freedom). The design of curved links of tensegrity mechanisms facilitates continuous change of the point of contact (along the curve) that results from the tip-over. This contrasts to the sudden and piece-wise continuous change for the case of robots with traditional straight links which generate impulse reaction forces during locomotion. The two resulting robots—the Icosahedron and the Sphericon Tensegrity Robots—display shape morphing (packing-unpacking) capabilities and achieve locomotion through internal mass-shifting. The presented static equilibrium analysis of sphericon with mass is the first step in the direction of dynamic locomotion control of these curved link robots.

## 1. Introduction

Tensegrity structures are comprised of disconnected rigid compressive elements (links) suspended by a network of pre-stressed tensile elements (cables). The redundant links impart robust and fault tolerance, the strategic prestressed cable-link combination provides them with compliance and shape morphing ability (packing-unpacking) (Skelton et al., 2001). These qualities have attracted considerable attention from roboticists to design tensegrity mobile robots for space and exploration applications (Paul et al., 2005, 2006; Shibata et al., 2009; Böhm et al., 2012, 2016; Khazanov et al., 2013; Bruce et al., 2014; Kim et al., 2014; Sabelhaus et al., 2015; Lin et al., 2016; Zappetti et al., 2017; Mintchev et al., 2018; Vespignani et al., 2018).

### 1.1. Tensegrity Prototyping

The geometrical analysis of tensegrity mechanisms has been substantially researched (Roth and Whiteley, 1981; Connolly and Back, 1998; Schenk, 2006; Skelton and de Oliveira, 2009). However, prototyping of tensegrity structures remains tedious and time-consuming (Kim et al., 2014; Chen et al., 2017a). This is due to the complexity of geometric morphologies that are challenging to visualize and requirement of prestress in the cables. Currently, the design methodologies utilize jigs, multiple sets of hands and precise fabrication to achieve symmetric cable tension and link compression (Böhm et al., 2016; Chen et al., 2017a; Kim et al., 2017; Cera and Agogino, 2018). Recently, planar-to-three-dimensional solutions have been explored using flexible lattice networks which are excellent for fabricating known morphologies which may not be altered post-assembly (Chen et al., 2017a; Zappetti et al., 2017). The compressive elements (links) are made of rigid material, including wood (Kim et al., 2014), plastics (Böhm et al., 2016), and metals (Paul et al., 2006; Sabelhaus et al., 2015; Chen et al., 2017a; Kim et al., 2017). The tensile and compliant elements are fabricated using cables, metal extension springs (Böhm et al., 2012; Khazanov et al., 2013; Bruce et al., 2014; Lin et al., 2016; Kim et al., 2017; Cera and Agogino, 2018) and elastic cables comprised of various plastics (Paul et al., 2006; Chen et al., 2017a; Zappetti et al., 2017; Mintchev et al., 2018). Springs may span the full cable length (Böhm et al., 2012; Khazanov et al., 2013), or pair in series with other cable materials (Bruce et al., 2014; Kim et al., 2017; Cera and Agogino, 2018).

### 1.2. Integration

Integration of these elements varies considerably, with some methods including hooks (Khazanov et al., 2013; Sabelhaus et al., 2015; Cera and Agogino, 2018), knots (Kim et al., 2014), and even clamps (Chen et al., 2017a). Here, precision in fabrication and integration of component lengths is critical to achieve the desired balance of forces required by the mechanism. Connections are often semi-permanent and restrict passive cable modification (a notable exception by Böhm et al., 2016). These limitations can be mitigated by active cable control (Kim et al., 2014, 2017; Sabelhaus et al., 2015; Vespignani et al., 2018). However, for tensegrity structures lacking active cable control, achieving even force distribution presents a challenge. A conventional solution is to determine the required component lengths of a structure before assembly. This solution is time-consuming and limits experimentation with novel morphologies.

### 1.3. Tensegrity Locomotion

Locomotion is a result of the optimization of frictional forces between the robot and its environment at different locations of the body (Radhakrishnan, 1998). In case of tensegrity robots, this is often achieved by altering the center-of-mass (CoM) of the robot to induce “tip-over” that subsequently results in change in the points of contact with the surface. In the case of traditional straight-link tensegrity robots, the change in points of contact (links and their corners) is sudden and results in impulse forces during “tip-over” sequences.

### 1.4. Contribution

The paper proposes a design methodology that employs modular and rapidly producible components, and is applicable to variable morphologies without requiring precise component proportions, prestressed cables, and use of jigs. The fabrication and integration solutions are utilized to design shape-morphing straight six-link Icosahedron and curved two-link Sphericon Tensegrity Robots that possesses packing-unpacking capabilities. For the latter robot, the alteration of the CoM through internal mass-shifting results in continuous change in points of contacts along the curved link. The static equilibrium analysis of one degree-of-freedom sphericon morphology as a function of position of weights is discussed.

## 2. Fabrication Methodology

**Figure 1:**
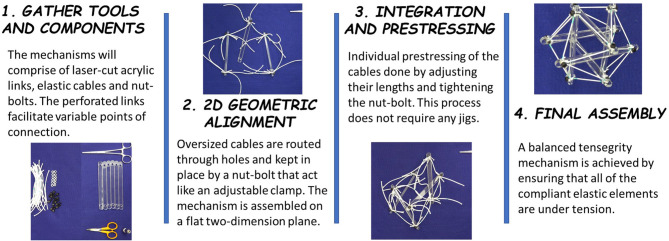
Assembling a tensegrity mechanism–(1) Perforated rigid links allow variable points of connection and nut-bolt combinations act as adjustable clamps. (2) Links are aligned on a planar surface and interconnected using oversized cables. (3) The cables are individually tensioned, without need of external jigs, (4) to construct the final balanced tensegrity mechanism.

Prototyping is critical for exploring geometric morphologies, prestresses, and other fabrication parameters of these mechanisms. The proposed methodology for tensegrity mechanisms assembly enables use of oversized tensile elements to support passive tuning of cable prestresses and is summarized in Figure 1 and Videos S1.

**Figure 2:**
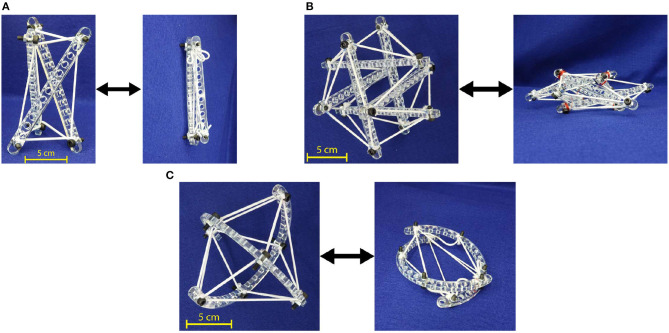
Straight and curved link tensegrity morphologies fabricated using the proposed methodology. **(A)** Three-link prism. **(B)** Six-link icosahedron. **(C)** Curved two-link sphericon.

The necessary components and tools include nuts and bolt, rigid links, elastic cables, scissors, and wrench. The quantity of nuts and bolts is equal to the number of intersections between links and cables in a given tensegrity design. For the prototypes discussed in this paper, the rigid links are laser-cut with a major dimension of 160 mm from 5 mm thick acrylic sheets and tensile cables are cut from 2 to 4 mm diameter elastic nylon cord. The perforated design of rigid, acrylic links facilitates variable points of connection on the links and prestress capability. The cables are routed through the proper holes in each link and are pinned in place by a bolt. The bolts used herein are M3-0.5 or M5-0.8 socket head cap screws. Every cable which passes through a given hole must be present before bolt placement. Bolts and holes are sized such that cables can be forced to change free length (tuned) but do not move during assembly. Cables are intentionally cut past their tuned free length so that routing to distant holes does not require stretching cables or forcing links into position. With sufficiently oversized cable lengths, all connections may even be made on a flat surface, eliminating the need for jigs (Figure 1). Finally, cables are adjusted to their desired free lengths by marking an expected free length on each cable and tuning from this far closer position. Once the model has reached an acceptable position, nuts are tightened on each bolt to prevent any possibility of cable shifting. The extra lengths of cable can be trimmed off to create a permanent structure or can be taped down to allow for later changes to clamping position. As illustrated in Figure 2, the presented methodology can fabricate of straight three-link prism which can be packed into a single combined link. Similarly, the straight six-link icosahedron and curved two-link sphericon can be packed into a planar sheet.

The proposed methodology provides the benefits of rapid prototyping and hassle-free assembly, and cable manipulation capability. The components are quickly produced, applicable to a range of designs, and simple to assemble. Cutting a link by laser takes around 2 min, while printing the same link through an FDM process takes 45 min (machine preparation times are approximately the same). Only five tools are used: fabric shears to cut cables, a BOSS LS-1416 laser cutter for the links, a wrench and hex key to modify the clamping force of the nut-bolts and forceps to grab difficult-to-grasp cables when tuning. Additionally, individual cables may be passively and independently clamped and removed without pretensioning. Icosahedron, as illustrated in Figure 2A, are assembled within an hour. This is approximately half of the fastest assembly time in the literature (Kim et al., 2014). The elastic lattice method proposed by Chen et al. (2017a) has yielded considerablly faster assembling (around 15 min) for a similar task that would require an hour and five people using conventional techniques. Nevertheless, this methodology does not allow in-place modification of the already assembled tensegrity mechanism or fabrication of unknown morphologies. Anzalone et al. (2017) have claimed to build a five-sided prism (a simpler shape) in 1 h, however, the fabrication methodology remains unspecified.

The three-link prism (Figure 2A) and the curved two-link sphericon (Figure 2C) are completed in approximately 10 and 25 min, respectively. Furthermore, individual cable tension and lengths may be passively modified during and after final assembly, enabling tunable levels of compliance within a structure.

## 3. Locomotion and Tensegrity Robots

### 3.1. Morphology Design for Locomotion

**Figure 3:**
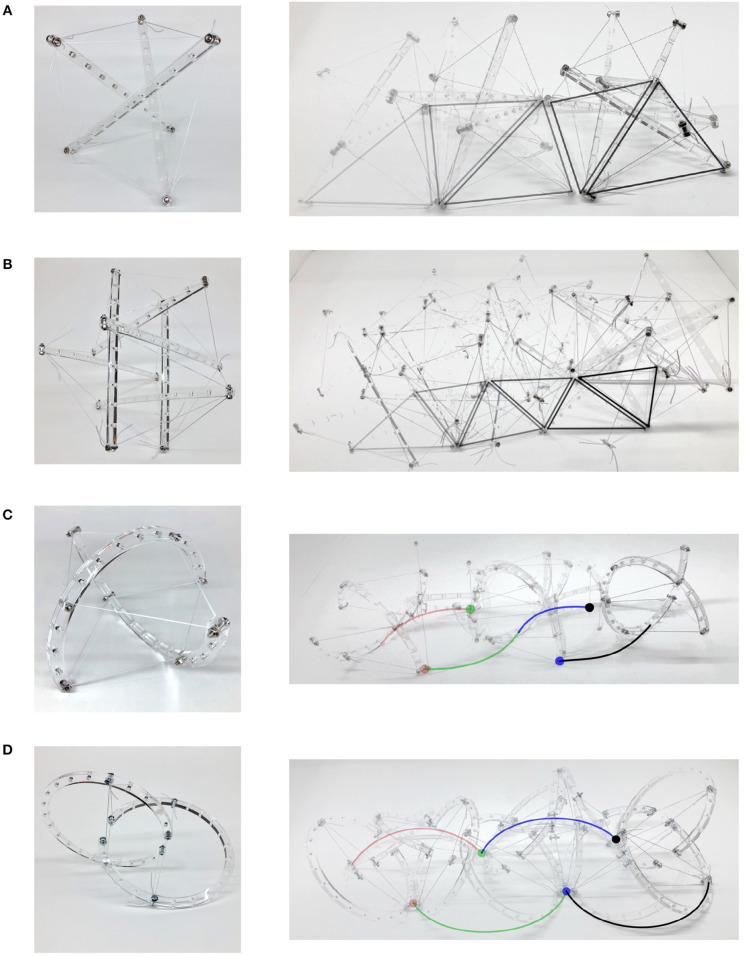
Locomotion for tensegrity mechanisms of different morphologies. For straight link mechanisms, the triangular faces contacting the ground are traced during motion. Likewise, the points of contact for curve link mechanisms are traced. **(A)** Highly puncuated zig-zag rolling motion for a structurally simple three-link prism. **(B)** Punctuated rolling motion along the faces of straight-link icosahedron resulting in continuous zig-zag. **(C)** Dual-axis wobble locomotion of two curve-link mechanism. **(D)** Uniaxial locomotion of oloid.

Tensegrity mechanisms adapted to mobile robots conventionally achieve locomotion through rolling about their body. Intuitively, morphologies resembling spheres facilitate smooth rolling which can be defined as continuous change in the point of contact along the body as the robot moves. Traditional straight link robots are limited in their ability to approximate a spheres curvature and achieve smooth rolling motion. Closer approximations to a sphere require increases in structural complexity, i.e., more links, cables and connections. For example, as shown in Figure 3A, a straight three-link tensegrity prism is notable for its design simplicity but not well-suited for rolling locomotion due to high discontinuity. In order to enhance rolling smoothness while optimizing structural complexity, the six-link icosahedron (Figure 3B) is frequently selected to achieve rolling locomotion (Shibata et al., 2009; Khazanov et al., 2013; Bruce et al., 2014; Kim et al., 2014; Lin et al., 2016; Cera and Agogino, 2018; Vespignani et al., 2018). This morphology enables planar locomotion, but motion is characterized by discontinuous “tip-over” impacts between triangular faces. Furthermore, these triangular faces are non-linearly sequenced, resulting in continuous “zig-zagging” directional change. This overall motion is described as “punctuated rolling motion” (Chen et al., 2017b) achieved through “steps” (Kim et al., 2017) or “flops” (Bruce et al., 2014). Mitigation of this problem may be achieved either by further increasing structural complexity (e.g., a 12-link rhombicuboctahedron morphology which is nearer to a sphere).

#### 3.1.1. Curve-Link Morphologies

Another approach to achieving smooth rolling locomotion is by directly introducing curvature to the compressive rigid links. Here, the curvature introduces additional bending moment to links (straining the definition of tensegrity). Smooth uniaxial rolling locomotion has been achieved using tensegrity mechanism comprising of two curved links—a morphology that resembles a condensed spehricon, rather than a sphere (Böhm et al., 2012, 2016; Kaufhold et al., 2017). A sphericon is a geometric roller formed by two orthogonal half-arcs meeting at the same center of curvature (Hirsch and Seaton, 2019) that is capable of uniaxial rolling. Furthermore, it has been observed that an improved geometric roller may be created through modification of these arc lengths and the distance between their respective centers of curvature (Kaufhold et al., 2017). This geometric roller may be adapted toward a tensegrity morphology capable of smooth uniaxial rolling and full planar locomotion with the addition of conventional “tip-over” operations (Böhm et al., 2016). These results invite further exploration into other curved link morphologies potentially suitable for tensegrity robot locomotion.

**Figure 4:**
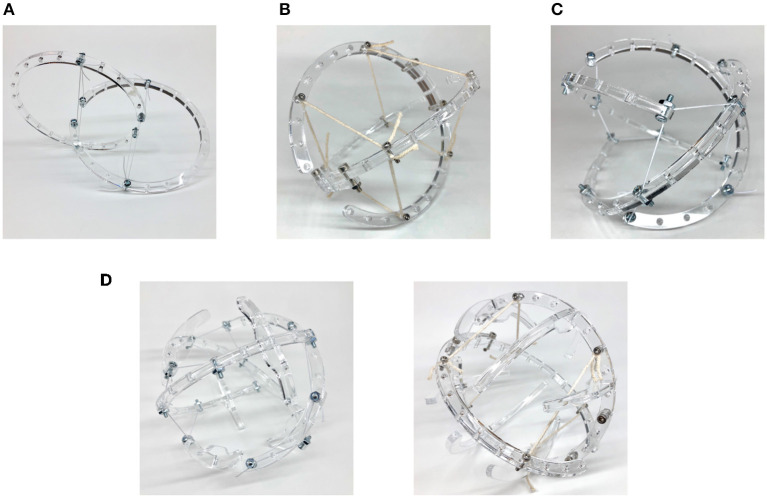
Exploring curve-link tensegrity morphologies. **(A)** Oloid. **(B)** Two disc roller. **(C)** Three curve-link. **(D)** Modified with non-structural features.

The curved two-link sphericon roller, as illustrated in Figure 2C, is a variation on Böhm-morphology (Böhm et al., 2016) where the centers of the arcs do not coincide. This was observed to have similar uniaxial locomotion with a slight wobble (Figure 3C). An oloid (Figure 4A) is also a uniaxial roller which further varies its arc angle beyond 180° which also demonstrates wobbly locomotion as shown in Figure 3D. The two-link shpericon roller (Figure 4B) is an oloid where the centers of the arcs coincide—it displays oloid-like locomotion along its outer edges which is hindered at its poles (ends of the curved links). The three-link morphology (Figure 4C) behaves similar to the straight three-link prism, i.e., inefficient but somewhat improved rolling locomotion. Adding non-structural curved features to these modified morphologies (Figure 4D) reduces wobble. These additional features function as an exterior shell for the structure, filling in portions of the open spaces between links but substantially compromising the packing ability of the modified mechanisms.

#### 3.1.2. Smooth Rolling of a Sphericon

**Figure 5:**
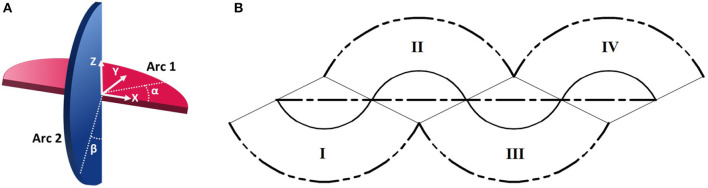
Periodic smooth locomotion of a sphericon. **(A)** Visualization of the two orthogonal arcs with associated angles of contact (α, β). **(B)** Paths of contact arcs (double-dashed line), center (continuous) and overall motion (dashed). Numerals denote the four distinct stages (quadrants) of the periodic motion.

When movement occurs without slipping, it is due to change in the points of contact between the mechanism and ground. Here, smooth rolling is defined as continuous change in these points of contact. The sphericon, illustrated in Figure 5A, demonstrates this quality where neither of the half-circular arc leaves contact with the ground as they trace the path shown in Figure 5B and Videos S3. During locomotion, the sphericon transitions between quadrants (wobbling motion) where the arcs smoothly trade roles—one provides the rolling contact surface (changing α or β) while the tip of other acts like a stationary contact point (α or β is 180° or 0°). The arcs with the fixed and varying points of contact are referred to as the stationary and rolling arc. The corresponding points of contacts are termed the stationary and rolling points of contact. For example, referring to Figure 5A, as α approaches 180° with β at 0° in quadrant I, the angle β begins to change from 0° while α is fixed at 180° in quadrant II. Table 1 summarizes the rotation direction and axis, and possible contact angles for each quadrant.

**Table 1:** Arc contact angles in each quadrant during smooth locomotion of a sphericon.

**Quadrant**	**I**	**II**	**III**	**IV**
Rotation axis	−*X*	+*Z*	+*X*	−*Z*
α	0° to 180°	180°	180° to 0°	0°
β	0°	0° to 180°	180°	180° to 0°

### 3.2. Tensegrity Robots

Controlled rolling locomotion in tensegrity robots is conventionally achieved by altering their CoM either through deformation of the body (Shibata et al., 2009) or internal shifting of the mass (Böhm et al., 2012, 2016). Through coordinated cable actuation, the body deformation results in change in the robots CoM and ground contact surface, causing the body to rotate. Here, actuating the large number of cables involved requires a sizeable amount of control effort. The internal mass-shifting strategy alters the robots CoM without deforming the body and facilitates smooth rolling locomotion. Furthermore, mass-shifting mechanisms only require a single actuator, and may be incorporated directly into existing links, independent of tensile cable networks. Consequently, this approach has been demonstrated to achieve high-speed locomotion with reduced control complexity and minimal actuation.

#### 3.2.1. Internal Mass-Shifting Mechanism

**Figure 6:**
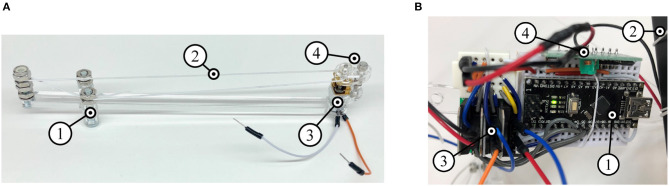
Design of the internal mass-shifting mechanism and control payload mechatronics are critical for design of an autonomous shape morphing tensegrity robot. **(A)** Pulley system composed of sliding mass holder (1), nylon cable pulley (2), motor (3), and acrylic gearbox (4). **(B)** Control payload consisting of a microcontroller (1), battery (2), motor drivers (3), and radio module (4).

**Figure 7:**
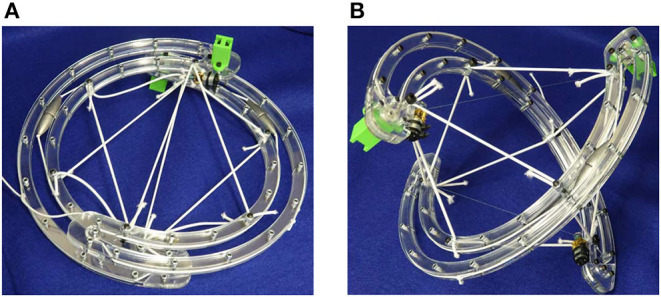
Folding of tensegrity robot achieved through motion of struts along the cables. **(A)** Packed orientation. **(B)** Deployed orientation.

The internal mass-shifting is achieved through a pulley system that can be directly integrated onto the links, enabling modular design of tensegrity robots capable of locomotion. Figure 6A illustrates the mass-shifting pulley system on a straight link. Here, the mass holder surrounds the link and is capable of sliding along it. The pulley cable (same as tensile cables) is attached to the mass holder which is fed through the gearbox of a motor at one end and looped around the other end. The gearbox is created out of laser-cut acrylic components and consists of a driving pinion and idler gear, which grip the cable as they rotate. The high torque gear motors provide a firm grip on the cable while both powered and unpowered. The current prototype, shown in Videos S2, uses a derivative of a Pololu Micro Metal Gearmotor. The pulley system is further adapted to modified curved struts as illustrated in Figure 7. Here, the pulley cable is inlaid inside the channels following the strut's curvature and the masses are directly held between the two curved sections while an end spool is employed to mitigate frictional forces.

#### 3.2.2. Control Payload

Available options for providing power and control to tensegrity robots present limiting factors in their design. A tethered robot may be simpler and lightweight, but is limited in range—either by the length of its cord, or the likelihood of tangling of the cord while rolling. Untethered robots require self-contained electronics, and potentially significant battery payloads. The presented control solution is created in pursuit of the minimum requirements of weight, size, and complexity to achieve a modular untethered system. Figure 6B illustrates the control payload that executes open-loop control commands wirelessly sent by an external controller.

#### 3.2.3. Shape Morphing for Packing and Deployment

Active folding of tensegrity robots has been achieved by the SUPERball tensegrity robot (straight six-link icosahedron) (Vespignani et al., 2018). This enables compact storage of tensegrity robots and subsequent active deployment which is highly desirable for space applications and disaster relief scenarios. Folding of these robots has conventionally been achieved through active cable length change (Bruce et al., 2014). An alternative method involving motion of link ends along cables is proposed as illustrated in Figure 7. The cables are fed through gearboxes (the same employed for mass-shifting) attached to motors at link ends. Folding is achieved by coordinating the motors at both link ends of the curved two-link sphericon robot.

Integration of the presented systems results in the creation of two mobile robots that are capable of locomotion through internal mass shifting—the Icosahedron (straight six-link) and Sphericon (curved two-link) Tensergrity Robots.

#### 3.2.4. Icosahedron Tensegrity Robot

**Figure 8:**
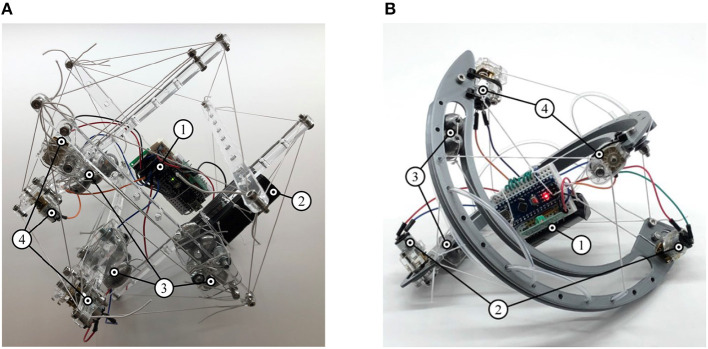
Tensegrity robots capable of locomotion through internal mass-shifting. They integrate (1) suspended control payload, (2) pulley system with (3) sliding masses, and (4) folding motors. **(A)** Icosahedron tensegrity robot. **(B)** Sphericon tensegrity robot.

The three orthogonal links were modified to incorporate mass-shifting systems and the electronics payload was distributed over two additional links as highlighted in Figure 8A. Locomotion challenges included optimizing the weight of the masses required for locomotion with the motor size and power. Mechatronics challenges arose from the scale of the morphology causing interference between the mass-shifting and control payload systems.

#### 3.2.5. Sphericon Tensegrity Robot

This morphology overcomes challenges faced by the previous case and incorporates mass-shifting systems into both curved links while the control payload was bundled and suspended in the center of the robot as illustrated in Figure 8B. Both these robots are shown in Videos S2. Consequently, a highly efficient locomotion is observed. By following the curvature of the robot, the masses are furthest from the geometric center of the robot and facilitate efficient altering of the robots CoM. The curved links enable smooth rolling motion by continuous change in points of contact with the variation of CoM. As the morphology only consists of two links, folding systems are incorporated without greatly increasing the required number of actuators showing considerable reduction in volume during packed orientation.

**Figure 9:**
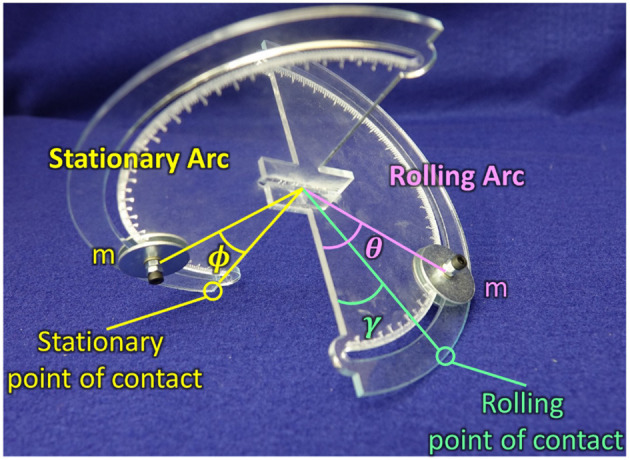
Sphericon mechanism with weights at static equilibrium position. The weights are located at angle θ, ϕ on the rolling and stationary arcs. The mechanism touches the surface at two points—stationary and rolling points of contact.

This tensegrity robot can be modeled as a sphericon mechanism (Figure 9). They are referred to as stationary and rolling arcs depending on how the point of contact changes with time. During dynamic motion as the sphericon rolls in one of the quadrants (Table 1), the stationary arc contacts the surface at the edges (α or β = {0°, 180°}) while the mechanism rolls along the rolling arc where as the point of contact dynamically varies along the arc (α or β ∈ [0°, 180°]). For example, when the shpericon travels in the first quadrant, the Arc 2 is the stationary arc where β = 0° and Arc 1 performs the role of the rolling arc as α varies between 0° and 180°. Here, two identical masses are located at angles θ and ϕ along the rolling and stationary arcs from the edges of the arcs.

**Figure 10:**
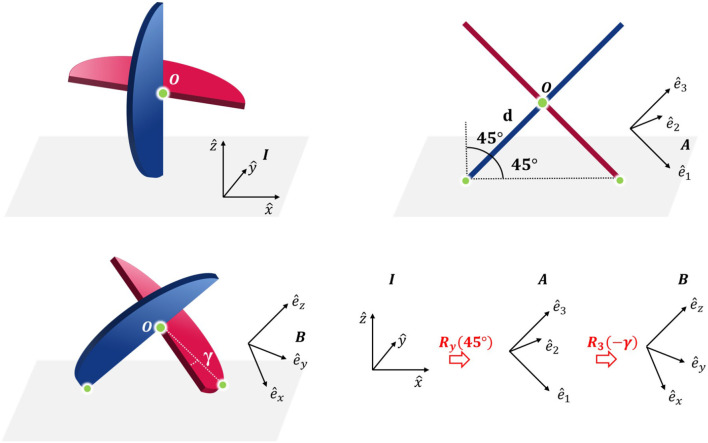
The relationship between the inertial (*I*), intermediate (*A*), and body coordinate system (*B*) of the sphericon. The point *O* indicates the center and the other two green points denote the points of contact between the mechanism and the surface.

Let *I* coordinate system be fixed in the inertial reference frame with origin at a point on a planar surface and orthonormal basis vectors $\left\{\hat{x},ŷ\right\}$ along the plane and ẑ normal to the plane. Similarly, let the *B* coordinate system be fixed on the body reference frame, origin at the center of the sphericon and orthonormal basis vectors {ê_*x*_, ê_*y*_, ê_*z*_}. The rotation matrix defining the relationship between the two coordinate systems can be written as (Craig, 1989)

(1) $${\;}_{B}^{I}R=R_{y}(45{}^{\circ})R_{3}(-\gamma )=\left[\begin{array}{lll} \text{ cos }\gamma \text{ cos }45{}^{\circ} & \text{sin }\gamma \text{cos }45{}^{\circ} & \text{sin }45{}^{\circ}\\ -\text{sin }\gamma & \text{cos }\gamma & 0\\ -\text{cos }\gamma \text{ sin }45{}^{\circ} & -\text{sin }\gamma \text{ sin }45{}^{\circ} & \text{cos }45{}^{\circ} \end{array}\right]$$

where the mechanism is rotated 45° about the y-axis of the inertial coordinate system *I* so each curve link rests on the ground (since the radii are equal this rotation is constant). Thereafter, it is rotated by −γ about the z-axis of the intermediate coordinate system (ê_3_) as illustrated in Figure 10. Consequently, the displacement vectors of the masses in the body *B* and inertial *I* coordinate systems are

(2) $$\begin{array}{ll} {\text{r}}_{O\to m1}=d{\left[\begin{array}{c} \text{cos }\theta \\ \text{sin }\theta \\ 0 \end{array}\right]}_{B} & =-\frac{d}{\sqrt{2}}{\left[\begin{array}{c} -\text{cos }\left(\gamma -\theta \right)\\ \sqrt{2}\text{ sin }\left(\gamma -\theta \right)\\ \text{cos }\left(\gamma -\theta \right) \end{array}\right]}_{I} \end{array}$$

(3) $$r_{O\to m2}=d{\left[\begin{array}{c} 0\\ -\text{sin }\phi \\ -\text{cos }\phi \end{array}\right]}_{B}\;=-\frac{d}{\sqrt{2}}{\left[\begin{array}{c} \text{cos }\phi +\text{sin }\gamma \text{ sin }\phi \\ \sqrt{2}\text{ cos }\gamma \text{ sin }\phi \\ \text{cos }\phi -\text{sin }\gamma \text{ sin }\phi \end{array}\right]}_{I}$$

Consequently, the velocity of these points can be calculated as

(4) $$\begin{array}{l} {\text{v}}_{mi}={\text{v}}_{O}+\frac{d}{dt}\left({\text{r}}_{O\to m_{i}}\right)\;\forall i\in \left\{1,2\right\} \end{array}$$

The potential energy of the system is equivalent to

(5) $$\begin{array}{l} V=m_{1}g\left({\text{r}}_{\text{O}}+{\text{r}}_{O\to m1}\right)\cdot \hat{\text{z}}+m_{2}g\left({\text{r}}_{\text{O}}+{\text{r}}_{O\to m2}\right)\cdot \hat{\text{z}}\\ \;=\left(m_{1}+m_{2}\right)g\frac{R}{\sqrt{2}}+m_{1}\text{cos }\left(\gamma -\theta \right)+m_{2}\left(\text{cos }\phi -\text{cos }\gamma \text{ sin }\phi \right)\; \end{array}$$

The static equilibrium positions of the mechanism can be obtained through the Lagrange's equations where the kinetic energy is zero and the generalized coordinate is γ

(6) $$\begin{array}{l} \frac{\partial V}{\partial \gamma }=0\to \tan\left(\gamma \right)=\tan\theta -\left(\frac{m_{2}}{m_{1}}\right)\cdot \left(\frac{\text{sin }\phi }{\text{cos }\theta }\right) \end{array}$$

## 4. Conclusion

The paper presents a design methodology for fabricating tensegrity robots of varying morphologies with modular components that facilitates rapid prototyping and hassle-free assembly, and capabilities to manipulate cable positions and tensions during assembly. Exploration of desirable morphologies for locomotion is critical to the design of tensegrity robots and includes investigation of their shapes (straight versus curved links), their placement (location of center of link arcs), number of links and even non-structural elements. The resulting two autonomous shape morphing tensegrity robots—the straight link Icosahedron and curved link Sphericon morphology—achieve locomotion through internal mass-shifting utilizing the presented mass-shifting mechanism. The curve link tensegrity robot demonstrates smooth locomotion and packing behavior with folding-deployment orientations.

## Data Availability Statement

All datasets generated for this study are included in the article/Supplementary Material.

## Author Contributions

TR fabricated all the tensegrity morphologies, designed the mechatronics, programming logic of the robots and contributed to the writing the draft. CG assisted TR with fabrication, writing and performed the mathematical analysis. VV supervised the research and oversaw all the activities.

### Conflict of Interest

The authors declare that the research was conducted in the absence of any commercial or financial relationships that could be construed as a potential conflict of interest.

## References

[B1] Anzalone P. Bayard S. Steenblik R. (2017). Rapidly deployed and assembled tensegrity system, in Acadia 2017 Disciplines & Disruption: Proceedings of the 37th Annual Conference of the Association for Computer Aided Design in Architecture (Boston, MA), 92–101.

[B2] Böhm V. Jentzsch A. Kaufhold T. Schneider F. Becker F. Zimmermann K. (2012). An approach to locomotion systems based on 3D tensegrity structures with a minimal number of struts, in 7th German Conference on Robotics, ROBOTIK 2012 (Munich), 1–6.

[B3] Böhm V. Kaufhold T. Schale F. Zimmermann K. (2016). Spherical mobile robot based on a tensegrity structure with curved compressed members, in 2016 IEEE International Conference on Advanced Intelligent Mechatronics (AIM) (Banff, AB), 1509–1514.

[B4] Bruce J. Caluwaerts K. Iscen A. Sabelhaus A. P. SunSpiral V. (2014). Design and evolution of a modular tensegrity robot platform, in 2014 IEEE International Conference on Robotics and Automation (ICRA) (Hong Kong), 3483–3489.

[B5] Cera B. Agogino A. M. (2018). Multi-cable rolling locomotion with spherical tensegrities using model predictive control and deep learning, in 2018 IEEE/RSJ International Conference on Intelligent Robots and Systems (IROS) (Madrid), 1–9.

[B6] Chen L.-H. Daly M. C. Sabelhaus A. P. Janse van Vuuren L. A. Garnier H. J. Verdugo M. I. (2017a). Modular elastic lattice platform for rapid prototyping of tensegrity robots, in Volume 5B: 41st Mechanisms and Robotics Conference (Cleveland, OH: ASME).

[B7] Chen L.-H. Kim K. Tang E. Li K. House R. Zhu E. L. (2017b). Soft spherical tensegrity robot design using rod-centered actuation and control. J. Mech. Robot. 9:025001 10.1115/1.4036014

[B8] Connolly R. Back A. (1998). Mathematics and tensegrity: group and representation theory make it possible to form a complete catalogue of strut-cable constructions with prescribed symmetries. Am. Sci. 86, 142–151.

[B9] Craig J. J. (1989). Introduction to Robotics: Mechanics and Control, 2nd edn. Boston, MA: Addison-Wesley Longman Publishing Co., Inc.

[B10] Hirsch D. Seaton K. A. (2019). The polycons: the sphericon (or tetracon) has found its family. *arXiv:1901.10677v2*.

[B11] Kaufhold T. Schale F. Bohm V. Zimmermann K. (2017). Indoor locomotion experiments of a spherical mobile robot based on a tensegrity structure with curved compressed members, in 2017 IEEE International Conference on Advanced Intelligent Mechatronics (AIM) (Munich: IEEE), 523–528.

[B12] Khazanov M. Humphreys B. Keat W. Rieffel J. (2013). Exploiting dynamical complexity in a physical tensegrity robot to achieve locomotion, in Advances in Artificial Life, ECAL 2013 (Sicily: MIT Press), 965–972.

[B13] Kim K. Agogino A. K. Moon D. Taneja L. Toghyan A. Dehghani B. (2014). Rapid prototyping design and control of tensegrity soft robot for locomotion, in 2014 IEEE International Conference on Robotics and Biomimetics (ROBIO 2014) (Bali: IEEE), 7–14.

[B14] Kim K. Moon D. Bin J. Y. Agogino A. M. (2017). Design of a spherical tensegrity robot for dynamic locomotion, in 2017 IEEE/RSJ International Conference on Intelligent Robots and Systems (IROS) (Vancouver, BC), 450–455.

[B15] Lin C. Li D. Zhao Y. (2016). Tensegrity robot dynamic simulation and kinetic strategy programming, in 2016 IEEE Chinese Guidance, Navigation and Control Conference (CGNCC) (Nanjing), 2394–2398.

[B16] Mintchev S. Zappetti D. Willemin J. Floreano D. (2018). A soft robot for random exploration of terrestrial environments, in 2018 IEEE International Conference on Robotics and Automation (ICRA) (Brisbane, QLD), 7492–7497.

[B17] Paul C. Roberts J. W. Lipson H. Cuevas F. J. V. (2005). Gait production in a tensegrity based robot, in ICAR, 12th International Conference on Advanced Robotics, 2005 (Belo Horizonte), 216–222.

[B18] Paul C. Valero-Cuevas F. Lipson H. (2006). Design and control of tensegrity robots for locomotion. IEEE Trans. Robotics 22, 944–957. 10.1109/TRO.2006.878980

[B19] Radhakrishnan V. (1998). Locomotion: dealing with friction. Proc. Natl. Acad. Sci. U.S.A. 95, 5448–5455. 9576902 10.1073/pnas.95.10.5448 PMC20397

[B20] Roth B. Whiteley W. (1981). Tensegrity frameworks. Trans. Am. Math. Soc. 265, 419–446.

[B21] Sabelhaus A. P. Bruce J. Caluwaerts K. Manovi P. Firoozi R. F. Dobi S. (2015). System design and locomotion of SUPERball, an untethered tensegrity robot, in 2015 IEEE International Conference on Robotics and Automation (ICRA) (Seattle, WA), 2867–2873.

[B22] Schenk M. (2006). Theory and design of statically balanced tensegrity mechanisms (Master's thesis). TU Delft, Delft, Netherlands.

[B23] Shibata M. Saijyo F. Hirai S. (2009). Crawling by body deformation of tensegrity structure robots, in 2009 IEEE International Conference on Robotics and Automation (Kobe), 4375–4380.

[B24] Skelton R. E. Adhikari R. Pinaud J. Helton A. J. W. (2001). An introduction to the mechanics of tensegrity structures, in Proceedings of the 40th IEEE Conference on Decision and Control (Cat. No.01CH37228), Vol. 5 (Orlando, FL), 4254–4259.

[B25] Skelton R. E. de Oliveira M. C. (2009). Tensegrity Systems. New York, NY: Springer.

[B26] Vespignani M. Friesen J. M. SunSpiral V. Bruce J. (2018). Design of SUPERball v2, a compliant tensegrity robot for absorbing large impacts, in 2018 IEEE/RSJ International Conference on Intelligent Robots and Systems (IROS) (Madrid), 2865–2871.

[B27] Zappetti D. Mintchev S. Shintake J. Floreano D. (2017). Bio-inspired tensegrity soft modular robots, in Biomimetic and Biohybrid Systems, Vol. 10384, eds Mangan M. Cutkosky M. Mura A. Verschure P. F. Prescott T. Lepora N. (Stanford, CA: Springer International Publishing), 497–508.

